# Proportionality between variances in gene expression induced by noise and mutation: consequence of evolutionary robustness

**DOI:** 10.1186/1471-2148-11-27

**Published:** 2011-01-26

**Authors:** Kunihiko Kaneko

**Affiliations:** 1 Department of Basic Science, Univ. of Tokyo, and Complex Systems Biology Project, ERATO, JST, 3-8-1 Komaba, Meguro-ku, Tokyo 153-8902, Japan

## Abstract

**Background:**

Characterization of robustness and plasticity of phenotypes is a basic issue in evolutionary and developmental biology. The robustness and plasticity are concerned with changeability of a biological system against external perturbations. The perturbations are either genetic, i.e., due to mutations in genes in the population, or epigenetic, i.e., due to noise during development or environmental variations. Thus, the variances of phenotypes due to genetic and epigenetic perturbations provide quantitative measures for such changeability during evolution and development, respectively.

**Results:**

Using numerical models simulating the evolutionary changes in the gene regulation network required to achieve a particular expression pattern, we first confirmed that gene expression dynamics robust to mutation evolved in the presence of a sufficient level of transcriptional noise. Under such conditions, the two types of variances in the gene expression levels, i.e. those due to mutations to the gene regulation network and those due to noise in gene expression dynamics were found to be proportional over a number of genes. The fraction of such genes with a common proportionality coefficient increased with an increase in the robustness of the evolved network. This proportionality was generally confirmed, also under the presence of environmental fluctuations and sexual recombination in diploids, and was explained from an evolutionary robustness hypothesis, in which an evolved robust system suppresses the so-called error catastrophe - the destabilization of the single-peaked distribution in gene expression levels. Experimental evidences for the proportionality of the variances over genes are also discussed.

**Conclusions:**

The proportionality between the genetic and epigenetic variances of phenotypes implies the correlation between the robustness (or plasticity) against genetic changes and against noise in development, and also suggests that phenotypic traits that are more variable epigenetically have a higher evolutionary potential.

## Background

Plasticity and robustness are basic concepts in evolutionary and developmental biology. Plasticity refers to the changeability of phenotypes in response to external environmental perturbations. Indeed many important concepts in biology are concerned with the changeability in the system. This changeability depends on each phenotype: some phenotypes are more variable than others. How is such degree of changeability characterized quantitatively?

On the other hand, robustness is another basic concept in evolutionary and developmental biology. Here, phenotypic robustness is defined as the ability of the system to continue to function despite perturbations to it [1-7]. Phenotypes important for survival are expected to be robust, at least to some degree, to enable organisms to survive under such perturbations.

For both plasticity and robustness, there are epigenetic and genetic sources of perturbations to a biological system, which act in different time scales. Epigenetic perturbation works at a faster scale. Phenotypes are changed through noise in gene expression and developmental dynamics. Environmental variation gives another source for variability. Genetic variation, on the other hand, works at a longer time scale through evolution. Now, is there any relationship between the changes of genetic and epigenetic origins? If a phenotype is changeable easily epigenetically through development or environment, is it also more feasible to change genetically? Similarly, if a phenotype is robust to developmental perturbations, is it also robust to genetic variations through evolution? When we consider generally any dynamical systems, such relationship would not be expected. However, as a biological system is a result of evolution, existence of some relationship between the genetic and epigenetic robustness may be expected.

The relationship between evolution and robustness has been long debated since the pioneering studies by Schmalhausen and Waddington [8,9]. Waddington, then coined the term "genetic assimilation", in which phenotypic changes induced environmentally are then assimilated to genetic changes through evolution. Although important, these pioneering studies have mostly emphasized the qualitative aspects of the relationship between robustness and evolution. However, advances in quantitative studies on cell biology have facilitated the quantitative assessment of this relationship. In particular, fluctuations of phenotypes (e.g., gene expression levels) that have been measured extensively through the fluorescent-protein techniques [10-13] can provide a tool to explore such relationship.

In quantitative terms, robustness can be considered as a measure of the insensitivity of phenotypes to external disturbances and plasticity as a measure of changeability of phenotypes. On the other hand, fluctuation is the degree of phenotypic variation induced by perturbations. Hence, the phenotypic fluctuation (variance) increases with a decrease in robustness, and vice versa. Thus, the variance can serve as an index (inverse) for robustness, and also for plasticity. Now, the question concerning robustness and evolution can also be posed in terms of phenotypic variances. How is the evolution speed correlated with the variance? Does the variance increase or decrease through evolution?

Indeed, findings of previous studies involving artificial selection experiments with bacteria suggest that the rate of evolution, i.e., the increase in the fitness per generation, is proportional to the phenotypic variance among isogenic individuals [14,15]. This relationship, originally defined on the basis of a macroscopic distribution theory, was also confirmed in in-silico experiments by using gene regulation networks (GRNs) and metabolic networks [16,17].

This observed relationship is noteworthy because evolvability is characterized by non-genetic variation of phenotypes. Even if the rate of genetic change (mutation or recombination) is identical, the rate of evolution can differ according to this variation. To elucidate this point, recall again that there are 2 sources in phenotypic variances, genetic and epigenetic. Quantitatively, the former is characterized by the phenotypic variance in a heterogenic population and is due to genetic modifications, as, denoted as *V_g_*, whereas the latter, denoted here as *V_ip_*, is the phenotypic variance in an isogenic population due to noise during the developmental process. The former reflects the structural robustness of the phenotype, i.e., the rigidity of the phenotype against changes induced by genetic mutations, whereas the latter reflects the robustness of the phenotype against the stochasticity encountered during the developmental process or that induced by environmental changes. (Phenotypic variance of non-genetic origin is traditionally discussed as environmental variance *V_e_*. Here, we are concerned with the variance due to fluctuation during developmental process, and thus adopt this notation, but one could regard this variance as a component of *V_e _*[18,19].)

It is obvious that evolution speed, which indicates the change in phenotype due to genetic changes, is correlated with *V_g_*, as was demonstrated by Fisher [18,20]. Thus, the proportionality between the evolution speed and *V_ip _*suggests the proportionality between *V_g _*and *V_ip _*throughout the course of evolution. Indeed, this relationship was confirmed by the evolutionary stability theory and numerical simulations [14,16], which imply that robustness to noise and to mutation are correlated.

So far the proportionality between *V_ip _*and *V_g _*of a given fitness *through the course of evolution (over generations) *was confirmed. Now, let us come back to the original question on a possible relationship between genetic and epigenetic robustness (or changeability) over many phenotypic traits. To discuss such problem, we need to study the relationship of the variances *V_ip _*and *V_g _*over many phenotypes or expressions of many genes, for a given individual (not over the evolutionary course). This is the focus of the present paper.

The phenotypes or gene expression levels are generally associated with several genes, as known as epistasis. Even if a fitness value may be directly related to a single phenotype or the expression of a single gene, many genes may modify the expression of each other. These expressions are interrelated through a complex gene regulation network; therefore, the nature of their correlation may change during the course of evolution. This may give some evolutionary constraint on the changes of expression levels of genes. Then, does a gene with a higher fluctuation in its expression level have a higher potentiality in evolution than others? Is there correlation between phenotypic changes by epigenetic noise in gene expression dynamics and by genetic variation? We will demonstrate such proportionality over expressions of many genes, rather than the previously studied proportionality of the variances through the evolutionary course.

Using a numerical model in GRNs and simulating their evolutionary changes required to increase a given level of fitness, we first found that the rate of evolution of the expression levels of several genes was highly correlated with (or roughly proportional to) the respective variances of these genes. Next, we proceeded to present evidence for the proportionality between the 2 types of variances of gene expressions, i.e., of genetic and epigenetic origins *over many genes*. This proportionality was achieved after a selection process under a given fitness condition and is true whenever the phenotype of evolved system is robust to transcriptional noise in gene expression dynamics and genetic mutation. Further, the generality of this relationship was confirmed by studying a variety of models and evolutionary stability theory of multivariate distribution. We will also discuss some experimental evidences for this relationship.

## Results

### Modeling Strategy

Here, we considered the evolution of a simple model for "development." For this purpose, we postulated the following conditions for development and evolution.

(i) The set of variables *x_i _*(*i *= 1, ⋯, *M *) represents the expression levels of *M *genes. These variables take continuous values, which we set such that if gene *i *is expressed, then *x_i _>*0 and if not, *x_i _<*0. (Choice of a threshold at zero is a matter of convenience. Indeed, we also carried out simulations of a model in which the expression *x_i _*takes only non-negative values with certain positive threshold values for activation. Results to be discussed were not altered by this change).

(ii) Gene expressions mutually activate or inhibit each other and are regulated by the GRN. The temporal evolution of the gene expression level *x_i _*is generally not simple because the value of *M *is large. The phenotype of each individual is defined by the set of gene expression level *x_i _*after evolution for "developmental time," which starts from the time point with a given initial gene expression level (set as *x_i _<*0 for all *i*, i.e., none of the genes is expressed). The developmental time for the system is set to a large period to allow gene expression patterns to settle into an attractor state.

(iii) Gene expression dynamics are noisy. Owing to their dependence on chemical reactions involving molecular collisions, gene expression dynamics are also stochastic in nature. In particular, since the number of molecules (e.g., mRNA and proteins) involved in gene expression is not extremely large, a deviation from the average rate equation for the reaction is possible. This deviation is represented as the noise applied to the average gene regulation expression dynamics. The amplitude of noise is denoted as *σ*.

(iv) Genotype: Depending on the genotype, the structure of GRN changes, i.e., network paths that determine the genes responsible for activating (or repressing) a given gene. This interaction between genes is represented by a connection matrix *J_ij_*, which takes a value of 1 (if activating), -1 (if repressing), or 0 (if there is no influence).

(v) Population: A population of individuals with different genotypes, i.e., with each individual having a slightly different GRN-the matrix *J_ij _*. This gives a genotypic distribution.

(vi) Fitness: The pattern of expression in *x_i _*is determined on the basis of gene expression dynamics. Fitness is a function of the expression pattern of a subset of *x_i _*, i.e., the expression of a given set of "target" genes *i *= 1, ⋯, *k*. If the pattern of *x_i _*is closer to the prescribed pattern of genes, the fitness is higher. We take the prescribed pattern as "all on" for the target genes, unless otherwise mentioned. If all components of *x_i _*(*i *= 1, ⋯, *k*) are positive, the fitness is set at 0, and it is decreased by 1 when the number of negative *x_i _*is increased by 1.

(vii) Mutation and selection: Offspring are produced according to the fitness: individuals with low fitness cannot produce offspring. The selection process according to the fitness thus progresses, while mutation introduces a change in the GRN, represented as 1,-1, or 0, in a few elements of the matrix *J_ij _*. The fraction of the elements altered is given by the mutation rate.

We studied numerical models based on the abovementioned characteristics. (For details see Method). Note that due to the complex nature of gene expression dynamics, GRN with a higher fitness value is generally rare. Further, since the expression dynamics are subjected to perturbations by noise, the "goal" of reaching the highest fitness may not be easily achieved. However, through the evolutionary processes, including natural selection and mutations, a GRN with the highest fitness value is generated. Individuals with the highest fitness value in a population evolve over some generations to reach the highest fitness value, i.e., *x_i _>*0, for the target genes *i *= 1, 2, ..., *k*.

We previously reported that the fitness distribution in a heterogenic population undergoes a transition when the noise level is increased [17,21]. Before we discuss the main results of the present paper from the next subsection, we briefly summarize the earlier numerical result in this subsection.

When the noise level *σ *was below a given threshold (*σ < σ_c_*), some individuals within the population might take lower fitness values, thereby inducing a considerable difference in the distribution of fitness over different genotypes. Some mutants derived from individuals with genotypes having high fitness values might take much lower fitness values, even after many generations of evolution. However, when the noise level was greater than the threshold (*σ_c _< σ*), the distribution of fitness was sharp, with concentration of mutants with high fitness values. Thus, low-fitness mutants were eliminated through the evolution. (If the noise level was too high, the expression levels were not fixed in time and increased or decreased over time. We did not examine such cases).

Accordingly, an increase in the noise strength lead to a transition in the robustness to mutation. GRNs that evolve under a low noise level did not have robustness to mutation, whereby some mutants could not sustain the high fitness value. In contrast, robustness to mutation was achieved for GRNs evolved only under a high noise level *σ > σ*.

This counterintuitive robustness to mutation for *σ > σ_c _*can be explained as follows. According to the dynamics of GRN that evolves under higher noise level, a large portion of the initial conditions reach target attractors that give the highest fitness values, thereby achieving robustness against noise, while for those evolved under *σ < σ_c_*, only a tiny fraction reaches target attractors. The developmental landscape for *σ > σ_c _*gives a global, smooth attraction to the target, whereas the landscape evolved at *σ < σ_c _*is rugged. Now, consider mutation to a network to slightly change gene expression dynamics. In the smooth landscape with global attraction, such a perturbation will cause little change to the final expression pattern, while under the dynamics with rugged developmental landscape, it often destroys the attraction to the target attractor. In other words, robustness to mutation evolves only under robustness to noise during development.

For *σ > σ_c_*, robustness to both noise and mutation evolved. We computed robustness to both factors using the 2 types of variances, *V_ip _*and *V_g_*, where the former was the noise-induced variance in the distribution of the fitness (i.e., the number of "on" target genes), in a population of isogenic individuals and *V_g _*is the variance in the distribution of fitness in a heterogenic population. The latter was obtained by first computing the mean fitness value for each genotype, and then by measuring the variance of these mean values for a heterogenic population. The 2 variances of the fitness decreased proportionally, throughout the course of evolution, for *σ > σ_c_*, in accordance with the principle of evolution of robustness (see [17,21]).

### *V_g_***-***V_ip _***relationship over genes**

Apart from the fitness level, the expression level *x_i _*of each gene *i *was also distributed, even for isogenic distribution with the same *J_ij_*; this was because of the stochasticity in each gene expression dynamics. Similar to the variances for the fitness, the phenotypic variance *V_ip_*(*i*) for each gene *i *in an isogenic population is defined on the basis of the variance of the expression of each gene *i*, with each *X_i _*= *Sign*(*x_i_*), in an isogenic population. On the other hand, the mean expression level $\bar{X_{i}}$ over the isogenic population depended on each genotype (i.e., the matrix *J_ij_*). The variance computed using the distribution of $\bar{X_{i}}$ in this heterogenic population, then, gives the genetic variance *V_g_*(*i*) for each gene *i*.

As mentioned above, our model also accounted for many non-target genes that do not contribute to fitness. The expression level *x_i _*of such non-target genes *i *could be either positive or negative because there was no selection pressure directed at fixing their expression level. However, we found that the expression levels of many non-target genes become fixed to positive or negative values over the course of evolution when *σ > σ_c_*. To achieve robust fitness, the expression levels of some of the non-target genes were also fixed to either the on or off status, with consistency across individuals in the gene expression status. Hence, the average expression level $<\bar{X_{i}}>$ in a heterogenic population increased or decreased to either positive or negative values ($\bar{...}$ is the mean over an isogenic population, while *< ... >*is the average over a heterogenic population).

**Figure 1:**
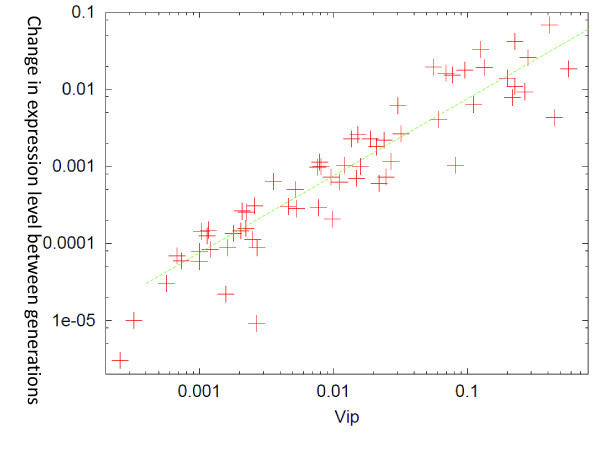
**Relationship between evolution speed and isogenic phenotypic fluctuations**. Following the Method, we computed the mean expression level $\bar{X(i)}=\bar{Sign(x_{i})}$ over 200 runs for each gene *i*. The ordinate shows the change of its average $<|\bar{X(i)}|>$ over all (= 200) individuals between the generations 80 and 90, representing the evolution speed of each gene expression *i*. The abscissa showed *V_ip_*(*i*), the variance of *X*(*i*) over 200 runs for the identical genotype (*J_ij_*). Computed as the variance at generation 80. The evolution progressed at around these generations and was completed before 200 generations. The noise level was *σ *= 0.08. See Method for details.

We computed the rate of evolution for each gene expression over a given number of generations, as the rate of either increase or decrease of the average expression level $<\bar{X_{i}}>$ in a heterogenic population. It was computed after evolution for some generation to achieve an increase in fitness. We then examined the validity of the relationship between the evolution speed and the fluctuations over genes, by plotting $|\;<\Delta \bar{X_{i}}>\;|\;$ against *V_ip_*(*i*), where $\;<\Delta \bar{X_{i}}>\;$ is the change in the average expression level $<\bar{X_{i}}>$ of a given gene over some generations (20, in Figure 1). The proportionality was found to be valid over different genes, both target and non-target, as shown in Figure 1. Genes with higher variances would have a higher potentiality to evolve (In Figure 1, the target genes had less variance and evolution speeds over these generations. This is because the fitness had already increased in this generation, fixing the expression levels to positive values, and there was little room for further increase).

**Figure 2:**
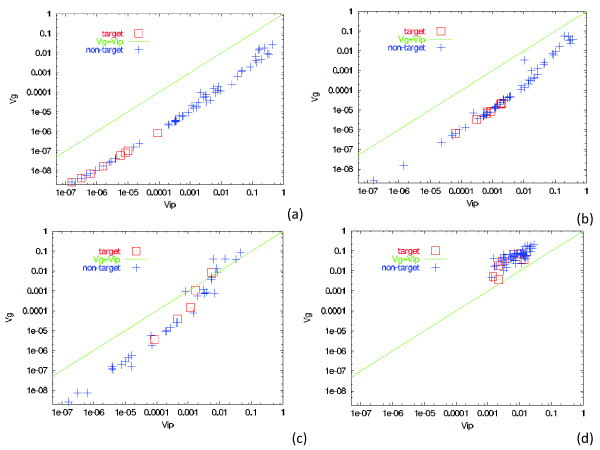
**Relationship between *V_g_*(*i*) and *V_ip_*(*i*)**. As described in the Method section of the text, *V_ip_*(*i*) was computed as the variance of the distribution of *Sign*(*x_i_*) over 200 runs for an identical genotype, while *V_g_*(*i*) was computed as a variance of the distribution of $\bar{\left(Sign(x_{i})\right)}$ over 200 individuals, where $\bar{Sign(x_{i})}$ was the mean over 200 runs. *σ *= 0.1 (a), 0.05 (b), 0.01 (c), and 0.001 (d). The plot of (*V_g_*(*i*) and *V_ip_*(*i*)) for all genes *i *at the 300th generation: Target genes as red ▢, and nontarget genes as blue +.

Considering the possible generalization of Fisher's theorem [18,20], which states that the evolution speed is proportional to *V_g_*, and applying it to the expression levels over genes, we may expect that the evolution speed of each gene expression level is proportional to *V_g_*(*i*). Then, the proportionality between *V_g_*(*i*) and *V_ip_*(*i*) over the genes can be expected from the proportionality between the evolution speed and *V_ip_*(*i*). Considering this, we examined the relationship between *V_g_*(*i*) and *V_ip_*(*i*) for several values of noise levels (Figure 2). From the plots, we obtained the following findings:

(i) Proportionality between *V_ip_*(*i*) and *V_g_*(*i*) was satisfied over many genes, i.e., *r_i _*≡*V_ip_*(*i*)*/V_g_*(*i*) took a common value *ρ*(*<*1) for many genes *i*, when the evolution of robustness progressed at *σ > σ_c_*.

(ii) Target genes always lay on the proportional line, with relatively low values of *V_ip_*(*i*) (and accordingly, *V_g_*(*i*)), while the variances of many non-target genes also lay on the same proportionality line *r_i _~ ρ*. Variances of only a few non-target genes did not exhibit the abovementioned proportional relationship, and the ratios *r_i _*for such genes were scattered between *ρ *and 1. (See also Additional file 1, Figure S1).

(iii) The fraction of such genes that fitted on the single proportional line increased with the noise strength *σ*. As the noise level was lowered, an increase was noted in the fraction of genes showing expression variances that deviate from the abovementioned proportional relationship. At around the threshold noise level *σ_c_*, most genes approached *r_i _~ *1. (See Additional file 1, Figure S1, for noise dependence of the variances *V_ip_*(*i*) and *V_g_*(*i*)).

**Figure 3:**
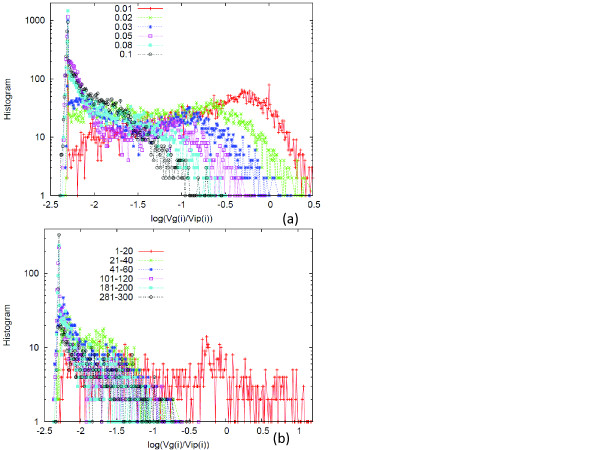
**Histogram of the ratio *V_g_*(*i*)*/V_ip_*(*i*)**. (a)The variances *V_g_*(*i*) and *V_ip_*(*i*) and their ratio were computed for each gene *i*, in the same manner as that adopted in Figure 2. Histogram of the $log_{10}\frac{V_{g}(i)}{V_{ip}(i)}$ was computed and sampled for all genes *i *from 280 to 300 generations. *σ *= .001 (red +), .02 (green x), .03(blue *), .05(pink ▢), and .08(sky blue *) and .1 (black ○). (b) Temporal evolution of the histogram for the ratio *V_g_*(*i*)*/V_ip_*(*i*) for *σ *= 0.1. The histogram was computed over all networks and for every 20 generations. We used the logarithmic scales for both axes, so that the values for abscissa shows $log_{10}\frac{V_{g}(i)}{V_{ip}(i)}$: The histograms over generations 1-20, 21-40, 41-60,101-120, 181-200, and 281-300 were plotted with a different color for each network. With evolution, a peak was formed at *V_g_*(*i*)*/V_ip_*(*i*) = *ρ *~ 10^-2.3^. See Method for the choice of the parameters.

We then plotted the histogram of *r_i _*over all genes *i*, sampled over a few sets of generations (see Figure 3a, plotted in log-scale for *r_i _*). The figure showed that the peak at *ρ *was more prominent with an increase in *σ*. On the other hand, the broader distribution ranging between *r_i _~ ρ *and 1 became more prominent as the noise level decreased, until the distribution around *r_i _~ *1 dominated at *σ ~ σ_c_*.

The proportionality between *V_ip_*(*i*) and *V_g_*(*i*) was not a property of every gene expression dynamics but was evident only after the system achieved robustness through evolution. The fraction of genes showing a proportional relationship increased during the course of evolution. Indeed, for *σ > σ_c_*, the peak at *r_i _~ ρ *increased over generations until it approached the distribution shown in Figure 3a (see Figure 3b). Summing up, the evolution of robustness was characterized by the formation of the peak at *ρ <*1, in the distribution of *r_i _*= *V_ip_*(*i*)*/V_g_*(*i*).

**Figure 4:**
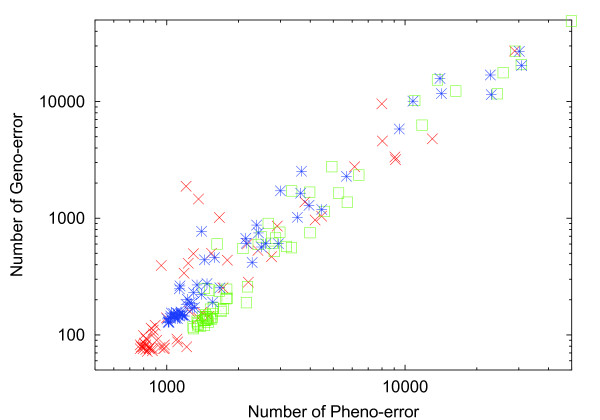
**Correlation between errors by noise and mutation over all genes**. The frequency of errors by mutation versus noise plotted over all genes for 3 networks evolved at low noise *σ *= .1 *> σ_c _*. The abscissa showed the number of events for which the expression of the gene is switched on or off by noise. Computation was carried out as follows: Taking an evolved network (with highest fitness), we simulated the gene regulation network(GRN) dynamics without noise for a sufficiently long duration and obtained the original value of *x*(*i*) for each gene *i*. (i) The GRN dynamics were then simulated in the same manner under the noise level *σ *= .1 for over 10^5 ^different runs, and the number of switch events (i.e., the runs in which *x*(*i*) changes its sign) was computed. (ii) We generated 10^5 ^networks by changing 50 paths randomly chosen from the original network, and simulated these GRNs without noise and computed the number of switch events for each gene *i*. Step (i) gives the number of error by noise, and step (ii) gives the error by mutation, for each gene *i*. The relations between these 2 errors were plotted for 3 original networks with a different color for each network.

The proportionality between the 2 variances implies the existence of a correlation between the noise- and mutation-induced changes in the gene expression statuses (see also Additional file 1, Figure S2 for the correlation in variances). Such a correlation was observed by computing the frequency of errors, i.e., changes in the on/off status of gene expression due to noise (without a change in the network) and as a result of mutation to the network (without adding the noise). The frequency of these 2 errors was highly correlated over genes for the GRN evolved at *σ > σ_c _*(see Figure 4). In other words, genes that were switched on or off more frequently by noise were also switched more frequently by mutation. This was in strong contrast with the GRN evolved at *σ < σ_c _*where no such correlation was observed (see Additional file 1, Figure S3). To sum up, the changeability of each gene expression level by noise and mutation was correlated, for a robust evolved system.

### Generality

We confirmed that the proportionality between phenotypic variances of genetic and epigenetic origins held true for a system with evolved robustness, by simulating our model and its extended versions over several conditions. For all the conditions below, we confirmed (a) transition to robust evolution with the increase in noise level (b) proportionality between *V_ip_*(*i*) and *V_g_*(*i*) throughout the course of evolution and over many genes *i*.

(i) Against the change in *k *(target set) and *M *(number of genes): With an increase in the fraction of target genes, evolution to the fittest state became increasingly dicult, and the noise level for robust evolution *σ_c _*was slightly increased, but the proportionality was valid for *σ > σ_c _*(see Additional file 1, Figure S4a).

(ii) Considering that the density in the connection paths in the actual GRN is rather low, the validity of the results was verified by decreasing the path rates in the model. The conclusion remained unchanged as long as the network was percolated. The fraction of genes deviating from the proportionality slightly increased as the path rate in the network decreased (e.g., to .05 per gene) (see Additional file 1, Figure S4b).

(iii) Even if the noise level depended on each gene *i*, the conclusion was valid (see Additional file 1, Figure S5). After evolution, the variance in the expression level of each gene was not correlated with the noise level of each gene, implying that the fluctuation of gene expression was mainly controlled by (evolved) gene-to-gene regulation *J_ij_*.

(iv) To consider the influence of extrinsic rather than intrinsic noise, we also introduced the same level of noise to all gene expressions. Again, the robustness and the proportionality between the variances persisted as long as the level of the intrinsic noise was larger than *σ_c_*. Although the "extrinsic" (common) noise also contributed to the evolution of robustness, it played only a minor role in this respect. (See Additional file 1, Figure S6).

**Figure 5:**
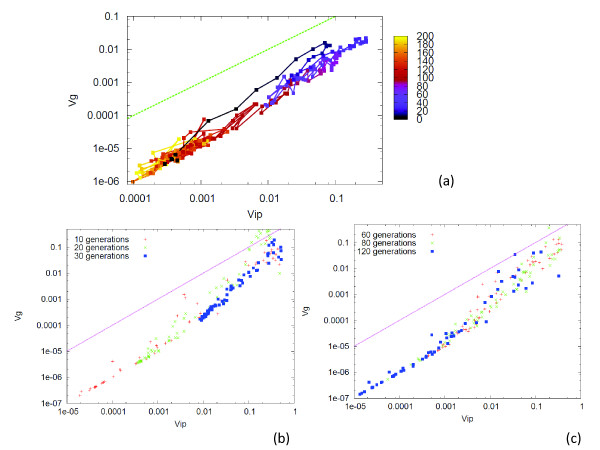
**Change in the variances after the switching of the fitness condition**. After evolution under the fitness condition to favor *x_i _>*0 ("on") for all the target genes *i *= 1, 2, ... *k*(= 8) as studied already, the fitness condition was switched at a certain generation to favor *x_i _>*0 for *i *= 1, 2, .., *k*/2 and *x_i _<*0 for *i *= *k/*2 + 1, .., *k *(i.e., the fittest gene expression pattern was given by + + + + *− − −−*, instead of + + + + + + ++). The switching was applied after a sufficiently large number of generations when the fittest networks are evolved (i.e., with *x_i _>*0 for the target genes). The switch initially caused a decrease in the fitness, but after a few dozens of generations, almost all networks evolved to adapt to the new fitness condition if *σ > σ_c_*. The values of the parameter and the procedure for computing the variances were identical with those in the previous cases. (i) The plot of the variances of the fitness, *V_g _*versus *V_ip _*per generation after the switching of the fitness condition. The color represented the generation number from the switching. There was a correlation between the increase in both the variances after the switch, and then, there was a proportional decrease as adaptation to the new fitness condition progressed. (b)(c) The plot of (*V_g_*(*i*) and *V_ip_*(*i*)) over all genes *i *at the generation 10, 20, and 30 for (b), and 60, 80, and 120 for (c). After the switch *V_g_*(*i*) and *V_ip_*(*i*) increased up to 30-60 generations, while the ratio *V_g_*(*i*)*/V_ip_(i)*approached unity for many genes. For generations *>*60, the variances decreased while the proportionality between *V_g_*(*i*) and *V_ip_(i)* was regained.

(v) By the variation in the environmental condition, the fitness condition for the target genes varied accordingly. By switching the condition for the target genes from 'on' to 'off' after some generations, we verified whether the evolution of GRN copes with this environmental variation. When the condition was switched, both the variances of epigenetic and genetic origins, as well as *r_i _*= *V_g_*(*i*)*/V_ip_*(*i*), increased to adapt novel environmental condition. Once the adaptation was achieved, the variances as well as *r_i _*decreased, to regain robustness (Figure 5).

When the target phenotype was changed periodically, we observed that the increase and decrease of the variances were consistently repeated, when the noise level was near the transition value (*σ_c_*), where rapid adaptation to new environment and robustness in phenotype were compatible.

(vi) We also extended our GRN model to account for diploids with sexual recombination. Here, each individual had a pair of matrices $J_{ij}^{1}$ and $J_{ij}^{2}$, and the gene expression dynamics were given as a result of summation of the two matrices instead of the equation in the Method. By considering recombination of two matrices from a parent, we evolved GRN to achieve a higher fitness. The proportionality of the 2 variances was again confirmed, while another noteworthy finding was that in the case of heterozygotes, the robustness was further enhanced (suppresses the variances of expression).

Further, the proportionality between the variances was not confined to the present model. Indeed, in a model of catalytic-reaction-network, such a proportionality between the variances of fitness [16] and over chemicals evolved, whereas robustness transition with the increase of noise was confirmed in an abstract spin model for protein folding [22]. We expect that the relationship holds true as long as the fitness is determined through complex developmental dynamics with noise and the high-fitness states are not easily achieved so that error catastrophe appears with the increase in the mutation rate.

### A phenomenological distribution theory

Considering the generality of the proportionality between the phenotypic variances of genetic and epigenetic origins, we provide a distribution theory for it without going into detailed setups of the model. We adopt the evolutionary stability argument first introduced for the proportionality between *V_ip _*and *V_g _*(of the fitness) through the course of evolution [16,23].

We consider a multivariate distribution function with regard to gene expression level *x_i _*and the genotype *a*. Considering that multiple genes are involved, we assume that the genotype is represented by a scalar parameter *a *(e.g., by a Hamming distance from the fittest genetic sequence). Now, we assume evolutionary stability, in which the distribution maintains a single peak through evolution. Then, by a suitable transformation of variables, this peak position is taken to be 0; further, the form is approximated by Gaussian distribution around the origin to give the following equation

(1) $$P(x_{i},\;a)=N_{0}exp\left(-\frac{x_{i}^{2}}{2\alpha_{i}}+C_{i}x_{i}a\;-\frac{a^{2}}{2\mu }\right).$$

with *N*_0_, a normalization constant so that ∫*P *(*x *: *a*)*dx *= 1. Here, *α_i _≡ V_ip_*(*i*) is the variance of the gene expression level, while *μ *is the mutation rate that determines the variance in the genotypes. Only a linear change of *x_i _*with regard to *a *is considered by neglecting higher order terms. Eq. (1) is rewritten as

(2) $$P(x_{i},\;a)=exp\left(-\frac{{\left(x_{i}-C_{i}a\alpha_{i}\right)}^{2}}{2\alpha_{i}}-\frac{1}{2}\left(\frac{1}{\mu }-C_{i}^{2}\alpha_{i}\right)a^{2}\right)$$

Now, recall the stability of the distribution *P *(*x_i_*, *a*), i.e., whether it has a peak in the space with *x_i _*and *a*. This condition is given by $1/\mu -C_{i}^{2}\alpha_{i}>0$, i.e., $\mu <\mu_{max}^{i}\equiv \frac{1}{C_{i}^{2}\alpha_{i}}$. For the mutation rate larger than $\mu_{max}^{i}$, the distribution is flattened. In this case, the peaked distribution concentrated at a certain gene expression level is no longer sustained. This can be interpreted as a kind of error catastrophe originally introduced by Eigen [24], i.e., collapse of the sustenance of the localized distribution of functional genes. The critical mutation rate for the error catastrophe is given by $\mu =\mu_{max}^{j}$, which can take independent values by any gene *i*. However, GRNs that have achieved robustness to noise and mutation through evolution may have some constraints among expressions of different genes.

To have higher robustness, the error threshold for the fitness should be postponed to a higher mutation rate. When the system has achieved robustness to noise and mutation through evolution, the fitness level changes only to a small degree (i.e., remains almost neutral) against a considerable amount of change in the GRNs due to mutation [21]. Until the occurrence of this number of mutations, the genes rarely undergo any change in their expression statuses (on or off). This introduces a constraint on the change in gene expression against mutations.

**Figure 6:**
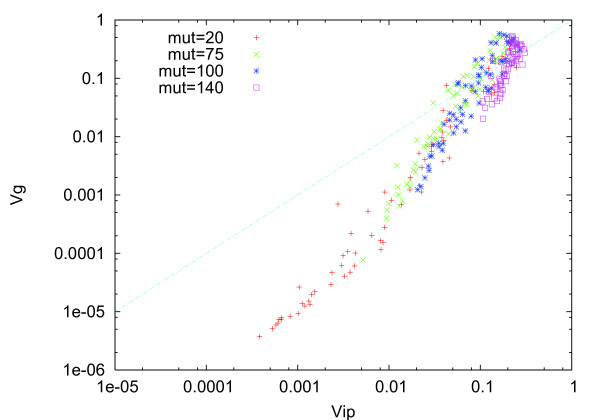
**Plot of (*V_ip_*(*i*), *V_g_*(*i*)) over all genes, after 200 generations, computed in the same manner as that described in Figure 2**. Instead of a single mutation per network adopted thus far, we used much a larger number of mutations. Each color represented the variances as a result of 20, 75, 100, and 140 mutations per network, which gave additions or deletions of paths chosen randomly in the gene regulation network (GRN). After mutation number ~ 100, many of genes touched the line *V_g_*(*i*) = *V_ip_*(*i*), where error catastrophe was percolated over many genes.

If each of non-target genes were switched on or off independent of each other, the error in the expression of target genes that could be influenced by each switch would occur frequently. Indeed, the evolved robust GRN has some constraint on the errors by noise and mutation, as plotted in Figure 4. To achieve higher robustness, there needs to be some correlation between the changes in the expression statuses of genes. By suitable mutual interaction (*J_ij _*≠ 0) among genes, the error frequency in the target gene expression can be reduced. This reduction works up to the mutation rate $\mu_{max}^{i}$, while for $\mu \sim \mu_{max}^{i}$, errors in the expression status of one gene can be propagated to the expression of many other genes, which in turn will induce changes in the expression statuses of other genes. Hence, for a robust network having higher error threshold mutation rate, many genes may be switched on or off simultaneously within a GRN, once an error catastrophe in one gene expression occurs. Accordingly, many genes *i *are expected to share a common critical mutation rate. Hence, $\mu_{max}^{i}$ is roughly equal for many genes, when robustness is evolved, i.e., at *σ > σ_c _*. In fact, we computed the expression pattern of GRNs by adding a larger number of mutations to the evolved GRN, to obtain the variances *V_ip_*(*i*) and *V_g_*(*i*). When the original network was evolved under high noise conditions, the variances touched with the line *V_ip_*(*i*) = *V_g_*(*i*) simultaneously over many genes, as the mutation rate was increased (see Figure 6). The flattening of the distribution occurred at similar mutation rates over many genes.

Following this argument, we may expect that

(3) $$\mu_{max}^{i}={\left(C_{i}^{2}\alpha_{i}\right)}^{-1}=independent\;of\;many\;genes$$

when robustness is evolved (i.e., at *σ > σ_c_*). Note that $\bar{x_{i}}$, the mean of *x_i _*for a given genotype *a*, is given by *C_i _α_i _a *according to eq. (2). The variance of $\bar{x_{i}}$ due to this "genetic change" is given by the distribution of *a*. Thus, we get

(4) $$V_{g}(i)=<{\left(\delta x_{i}\right)}^{2}>=C_{i}^{2}\alpha_{i}^{2}<{\left(\delta a\right)}^{2}>.$$

Since *V_ip _*(*i*) = *α_i _*, we get

(5) $$r_{i}=V_{g}(i)/V_{ip}(i)=C_{i}^{2}\alpha_{i}<{\left(\delta a\right)}^{2}>,$$

but this value is independent of gene *i*, according to eq.(3). Hence, the proportionality over genes is explained by a common error catastrophe threshold value over different gene expression levels.

As stated earlier, this proportionality over genes is not a general property of (gene expression) dynamical systems, but emerges only when evolution occurs under a sufficient level of noise (see Figure 3b). The use of the distribution function and assumption of stability implies that we are concerned with the stationary distribution that is attained after the progress in evolution. As the noise level reduces resulting in a decrease in robustness, the fraction of genes sharing a common value of *V_g_*(*i*)*/V_ip_*(*i*) = *ρ *decreases. This decrease is interpreted as a decrease in the number of genes that have a common error threshold value.

## Discussion

The relationship between the variances over genes, rather than the evolutionary course, will be easier to confirm experimentally because variances in this case need not be traced across many generations. By measuring directly the isogenic phenotypic variance and mutational variance over many genes (proteins), the correlation between the 2 can be examined. Although direct experimental support is not yet available, recent studies conducted by Laundry et al. [25] on such variances in yeast suggest the existence of a correlation between the 2 types of variances (see also [26]). In fact, they measured "expression noise" for each gene as the variance from its expression in isogenic organisms, and "mutational variance" as the variance of the change in the expression levels of the genes after the occurrence of mutations. The former corresponds to *V_ip_*(*i*), while the latter correlates with *V_g_*(*i*) since both measure the variations in phenotype (gene expression) induced by genetic changes. Although experimental data are scattered, a positive correlation is noted between the expression noise and mutational variance, as is consistent with the inference of the proportionality between *V_ip_*(*i*) and *V_g_*(*i*). Note that this proportionality holds true only for a set of genes whose expression levels are mutually related and directly or indirectly related with the fitness, whereas the experimental data cover all genes. In fact, by choosing a set of genes that have a stronger mutual relationship, the correlation between the variances is increased [27].

According to the theoretical argument we presented here, the phenotype variable is not restricted to gene expression level, but can represent any trait. Stearns et al. carried out selection experiments of *Drosophila melanogaster *on several fitness conditions such as age at eclosion, weight, and lifespan. They measured the variance of these phenotype traits within lines (i.e., corresponding to *V_ip_*(*i*)) and among lines (corresponding to *V_g_*(*i*)). Interestingly, the 2 types of variances (even after being normalized by the mean) showed remarkable proportionality over different phenotypic traits (see Figure 2 of [28]), in agreement with our theory. Note that they used the population selected on the basis of certain phenotypic traits, as in our model and theory. This must explain why the observed proportionality between the 2 variances is clearer than that in [25].

## Conclusion

The characterization of robustness, evolvability, and plasticity [1,29-31] is an important issue in the field of evolutionary and developmental biology; however, studies on this issue are often qualitative. In the present study, we have demonstrated that the phenotypic fluctuations provide quantitative measures for these. Consider a population of organisms evolved under a single fitness condition, where the phenotypes that directly or indirectly influence the fitness are given as a result of (gene expression) dynamics under noise (determined by transcriptional networks). Through selection and mutation, the rules for the dynamics (i.e., the transcriptional networks) evolve leading to the achievement of a higher fitness level. Previously, we defined *V_ip _*as the variance of the fitness within an isogenic population, and *V_g _*as the variance of the average fitness within a heterogenic population, and obtained *V_ip _*∝ *V_g _*∝ evolution speed through the course of evolution [16,17,23].

In the present paper we defined the variances at each gene expression level *i *due to noise as *V_ip_*(*i*) and that due to mutation as *V_g_*(*i*). Then, the conclusion of the present paper is summarized as follows:

(1) For a population of organisms at a given generation evolved after some generations, *V_ip_*(i) ∝ *V_g_*(*i*) for most genes *i*. In other words, *r_i _*= *V_g _*(*i*)*/V_ip _*(*i*) took a common value (*ρ <*1) over many genes, and the number of such genes increased as the robustness of fitness increased. (Total phenotype variance is given by *V_ip_*(*i*) + *V_g_*(*i*) if the 2 variances are added independently. In this case the heritability [18,19], defined as the ratio of *V_g_*(*i*) to the total phenotypic variance is given by *r_i_/*(1 + *r_i_*). Hence, the heritability takes a common value for mutually correlated traits, for evolved population under a fixed, single fitness condition). The previous relationship through the evolutionary course, implies that organisms with larger phenotypic variances have higher rates of evolution. The relationship (1) we found here implies that genes (or phenotypic traits) that have larger fluctuations have higher evolution speed.

(2) As the fraction of genes sharing a common value *r_i _*= *ρ <*1 increased, there was a decrease in the degree of freedom to change the gene expressions independently by mutation. This increase in correlated change lead to an increase in robustness of the fitness to mutation and noise. On the other hand, the expression of genes with larger *r_i _*~ 1 was easily switched by noise or mutation, and provided plasticity to environmental as well as mutational change (see also Figure 5).

The generality of our results was confirmed by several extensions of the model including environmental fluctuations, gene-dependent noise amplitudes, diploid with recombination, and so forth, as well as a catalytic reaction network model. Here, we should note that a correspondence between the change induced by noise in development and that induced by mutation was a source of correlation between *V_ip _*and *V_g _*in our study. Indeed, Waddington coined the term "genetic assimilation" as a process in which environment-induced phenotypic changes are subsequently embedded into genes [9]. The proportionality among phenotypic plasticity, *V_ip_*(*i*), and *V_g_*(*i*) is regarded as a quantitative expression of this genetic assimilation.

## Method

A simplified gene expression dynamics with a sigmoid input-output behavior [32,33] is adopted here, although several simulations in the form of biological networks will give essentially the same result. In this model, the dynamics of a given gene expression level, *x_i_*, is described by the following:

(6) $$dx_{i}/dt=\gamma \left\{\tanh\left[\beta \sum_{j>k}^{M}J_{ij}x_{j}\right]-x_{i}\right\}+\sigma \eta_{i}(t),$$

where *J_ij _*= *−*1, 1, 0, and *η_i _*(*t*) is a Gaussian white noise given by *< η_i _*(*t*)*η_j _*(*t'*) * >*= *δ_i, j _δ*(*t − t'*). *M *is the total number of genes, and *k *is the number of target genes that determine fitness. The summation only for *j > k *is introduced to eliminate possible influences from the target genes, which might also fix other gene expressions. Without this restriction and just by the summation over all genes, however, conclusions of the present numerical results are invariant. Of course, the matrix *J_ij _*is generally asymmetric. The amplitude of noise strength is given by *σ *that determines stochasticity in gene expression. The initial condition is given by (-1,-1,...,-1); i.e., all genes are off. The fitness *F *is determined by how many of the "target" genes are on after a sufficient time, i.e., the number of *i *such that *x_i _>*0 for *i *= 1, 2, ..*k < M*. Because the model includes a noise component, the fitness can fluctuate at each run, which leads to a distribution in the fitness *F *and *x_i _*, even among individuals sharing the same gene regulation network. For each network, we compute the average fitness $\bar{F}$ over *L *runs.

At each generation, there are *N *individuals with slightly different *J_ij_*. Among the networks, we select those with higher fitness values. From the selected networks, *J_ij _*is "mutated," i.e., *J_ij _*for a certain pair *i, j *selected randomly with a certain fraction is changed among ± 1,0. For example, each of the *N_s_*(*< N *) networks with higher values $\bar{F}$ produce *N/N_s _*mutants. (We also used the selection procedure such that the offspring number is proportional to the fitness, with normalization of the total population to *N*, but the same results presented here were obtained). We repeat this selection-mutation process over generations.

The variance of fitness or gene expression *Sign*(*x_i_*) of identical networks over *L *runs gives *V_ip _*or *V_ip_*(*i*), while the variances of their mean over different networks *J_ij _*give *V_g _*or *V_g_*(*i*). we chose *N *= *L *= 200, and *N_s _*= *N/*4, while the conclusion to be shown below does not change as long as these values are sufficiently large. We use *β *= 7, *γ *= .1, *M *= 64 and *k *= 8, and initially chose *J_ij _*randomly with equal probability for ± 1,0, unless otherwise mentioned.

## Supplementary Material

Additional file 1 **Figure S1 Dependence of the variances *V_ip_*(*i*) and *V_g_*(*i*) on the noise strength**. **Figure S2 **Correlation between gene expressions. **Figure S3 **Correlation between errors by noise and mutation over all genes. **Figure S4 **Relationship between *V *(*i*) and *V_ip_*(*i*) for evolved GRNs with a larger fraction of target genes, and a smaller fraction of nonzero genes. **Figure S5 **Relationship between *V_g_*(*i*) and *V_ip_*(*i*) for the gene expression dynamics whose noise level depends on each gene. **Figure S6 **Relationship between *V_g_*(*i*) and *V_ip_*(*i*) for a model with "extrinsic noise." Click here for file
