# Correction: Proportionality between variances in gene expression induced by noise and mutation: consequence of evolutionary robustness

**DOI:** 10.1186/1471-2148-12-240

**Published:** 2012-12-09

**Authors:** Kunihiko Kaneko

**Affiliations:** 1 Department of Basic Science, Univ. of Tokyo 3-8-1 Komaba, Meguro-ku, Tokyo 153-8902, Japan

## 

Although the simulation data as well as the conclusion on the proportionality between *V*_*ip*_(*i*) and *V*_*g*_(*i*) in the work [1] is correct, interpretation of some data therein should be corrected. As the sampling number (*L *= 200) to measure the average gene expression level is not large enough, there is a bias in the estimate in *V*_*g*_(*i*). Finiteness in the number of sampling *L* will generally cause a bias of the order of *V*_*ip*_(*i*)/*L*, in the estimate of the variance *V*_*g*_(*i*). The too good proportionality between *V*_*ip*_(*i*) and *V*_*g*_(*i*) for large *σ*, shown in Figure two (a)(b) of [1] (especially for small *V*_*g*_(*i*)), is due to this artifact. Accordingly, the sharp peak at ∼1/*L *= 1/200 in Figure three of [1] is due to this insufficiency by the sample number.

Still, the proportionality between the two variances *V*_*ip*_(*i*) and *V*_*g*_(*i*), albeit not so sharp, holds, as already observed in the region with larger *V*_*g*_(*i*) in [1]. We have simulated the model with a larger number of samples, i.e., *N *=* L *= 1000. As is shown in Figure 1, the proportionality is well discernible, where the proportion coefficient *V*_*g*_(*i*)/*V*_*ip*_(*i*) decreased with the increase in the noise level *σ*, which was already observed in the broad peak beyond 1/*L* in Figure three of [1]. This broad peak beyond 1/*L *in Figure three of [1] was found to be sharper as *N* was increased, from 200 to 1000. This peak indeed corresponds to the proportion coefficient extracted from Figure 1 in the present Correction. As the noise level *σ* was increased, the peak position *ρ *=* V*_*g*_(*i*)/*V*_*ip*_(*i*) decreased. Hence for larger *σ*, larger *L* is needed to get reliable estimate in the proportion coefficient. As for Figure five and Figure six of [1], the sharp proportionality for *V*_*g*_(*i*) ≲ 0.001 is due to the above bias, while the discussion therein concerns with the approach of *V*_*g*_(*i*) to *V*_*ip*_(*i*) at larger *V*_*g*_(*i*), which is not affected by the bias here.

**Figure 1:**
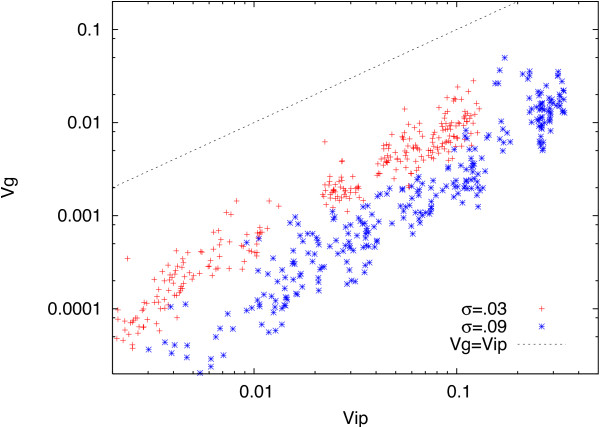
**Relationship between *V***_***g***_**(*i*) and *V***_***ip***_**(*i*).** As described in the Method section of [1], *V*_*ip*_(*i*) was computed as the variance of the distribution of *Sign*(*x*_*i*_) over *L* runs for an identical genotype, while *V*_*g*_(*i*) was computed as a variance of the distribution of $\left(\bar{\text{Sign}(x_{i})}\right)$ over *N* individuals, where $\bar{\text{Sign}(x_{i})}$ was the mean over *L* runs. Here we adopted *N *=* L *= 1000, instead of 200 in [1]. *σ *= 0.09 (blue *) and 0.03 (red +). The plot of (*V*_*g*_(*i*) and *V*_*ip*_(*i*)) for all genes *i* over 55-65th generations, where we have plotted only those genes with *V*_*g*_(*i*) > .0002, as the those with smaller than that may have little accuracy in estimating *V*_*g*_(*i*).

To sum up, the main claim of [1], i.e., proportionality between *V*_*ip*_(*i*) and *V*_*g*_(*i*) is valid, but the value of the proportion coefficient *ρ *=* V*_*g*_(*i*)/*V*_*ip*_(*i*) should be corrected. It decreases with the noise level, in contrast to the discussion in [1] for large *σ*. Major factor on this proportionality is attributed to the correlation of each variance with the average value $\bar{\text{Sign}(x(i))}$: In other words, a state with an intermediate expression level (i.e., smaller $\left|\bar{\text{Sign}(x(i))}\right|$) can be more easily switched on or off, both by noise and also by mutation, and hence the variances generally increase as $\left|\bar{\text{Sign}(x(i))}\right|$ approaches 0. Still, some correlation between *V*_*ip*_(*i*) and *V*_*g*_(*i*) remains even after removing this correlation through $\bar{\text{Sign}(x(i))}$.

I regret any inconvenience that misintepretation of the data with an insufficient sample size may have caused.
