# Supplementary material for: Proportionality between variances in gene expression induced by noise and mutation: consequence of evolutionary robustness
Source: BMC Evol Biol. 2011 Jan 26;11:27. doi: 10.1186/1471-2148-11-27 (PMC3045907; doi:10.1186/1471-2148-11-27)
Supplement: Additional file 1 — Figure S1 Dependence of the variances Vip(i) and Vg(i) on the noise strength. Figure S2 Correlation between gene expressions. Figure S3 Correlation between errors by noise and mutation over all genes. Figure S4 Relationship between V (i) and Vip(i) for evolved GRNs with a larger fraction of target genes, and a smaller fraction of nonzero genes. Figure S5 Relationship between Vg(i) and Vip(i) for the gene expression dynamics whose noise level depends on each gene. Figure S6 Relationship between Vg(i) and Vip(i) for a model with "extrinsic noise." [file 1471-2148-11-27-S1.PDF]

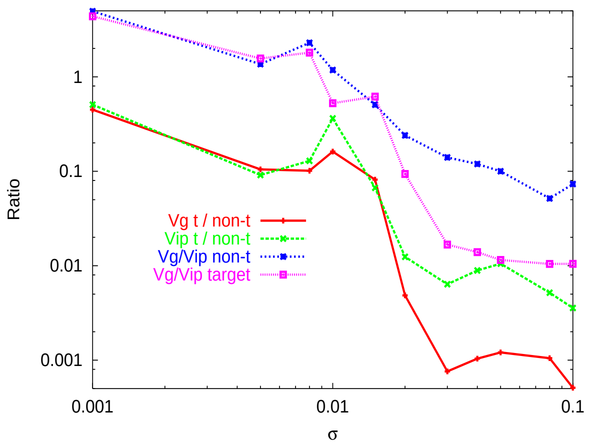

Fig. S1 Dependence of the variances $V_{ip}(i)$ and $V_g(i)$ on the noise strength. We considered networks with the gene regulation dynamics described in the text; these networks evolved under a given noise strength, σ. The variances were computed over all genes, i. The average of the variances $V_{ip}(i)$ and $V_g(i)$ were computed separately for target genes and non-target genes and plotted as a function of σ. The dependence of the variances was computed as an average for over (200) networks undergoing evolution for 280-300 generations.

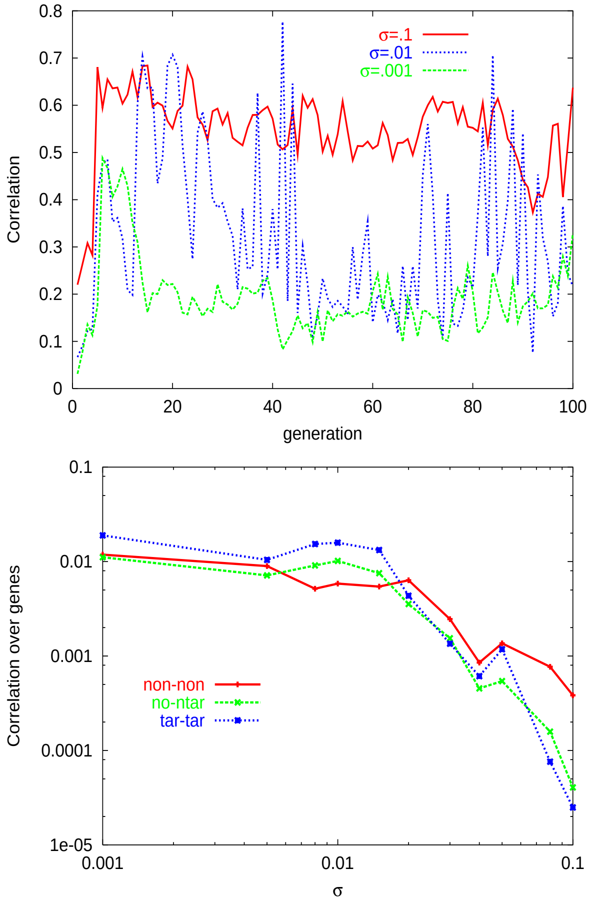

Fig. S2: (a) Temporal evolution of the correlations between genetic and epigenetic fluctuations. A characteristic feature of gene expression dynamics with evolved robustness was the correlation between the fluctuations of genetic and epigenetic origins. We measured $<\delta X_i \delta X_j>$ by separating it into 2 parts: one, representing genetic changes due to mutation and the other, epigenetic changes due to transcriptional noise. We found that the correlation between the 2 fluctuations increases with the progress of the evolution robustness. (b) Correlation between gene expressions: It might be expected that simple correlations $<\delta X_i \delta X_j>$ between 2 different gene expression levels increased such that all the fluctuations were highly correlated. In fact, in spite of the existence of the constraint imposed on gene expressions, this was not the case. We plotted the average of this correlation, while separately considering the correlations for target-target, nontarget-nontarget, and nontarget-target. This average was plotted as a function of the noise strength σ. The correlation of fluctuations in gene expression $<\delta X_i \delta X_j>$ was indeed smaller when evolution progressed to achieve robustness at $\sigma > \sigma_c$.

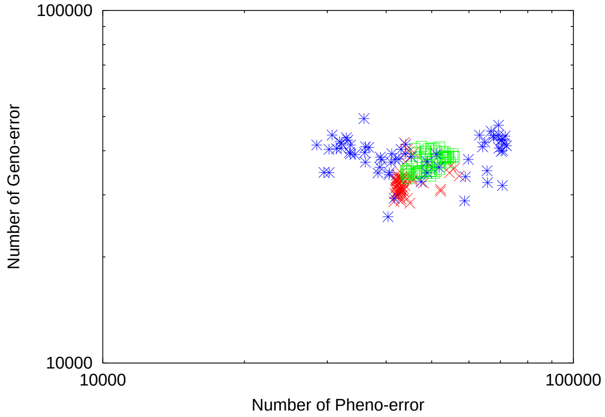

Fig. S3: Correlation between errors by noise and mutation over all genes. For comparison with Fig.4, the frequency of errors by mutation versus errors by noise for all genes was plotted, for 3 networks evolved under low level of noise $\sigma = .001 < \sigma_c$, where the evolved network did not have robustness to mutation. We followed the algorithm given in the caption of Fig.4, with a few changes: the noise level was considered as 0.001, and the number of paths changed by mutation was taken as 1 instead of 50 because evolved networks often underwent switches in gene expression even by a single-point mutation. As shown, no correlation was observed between the 2 error frequencies, in contrast to Fig. 4.

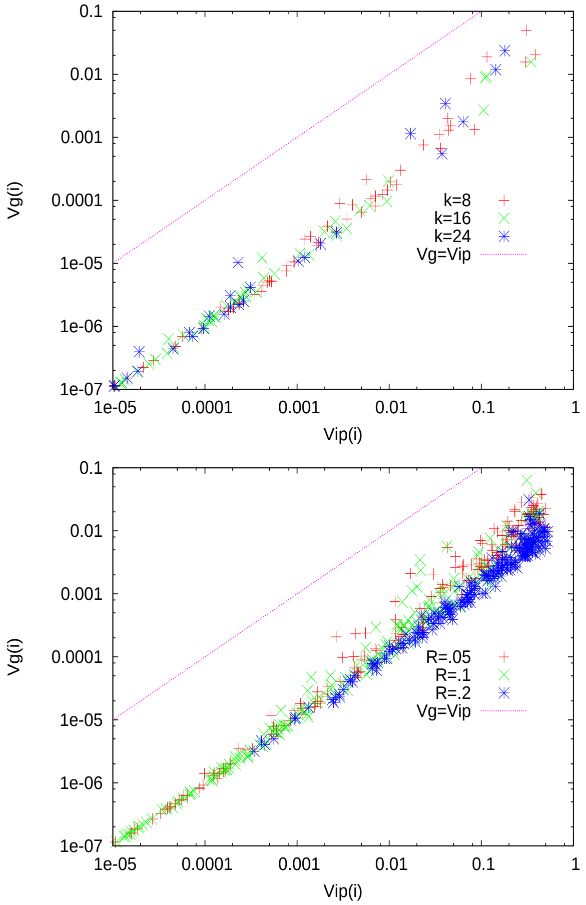

Fig. S4: (a)Relationship between $V_g(i)$ and $V_{ip}(i)$ for evolved GRNs with a larger fraction of target genes. $V_g(i)$ and $V_{ip}(i)$ were computed by the same procedure described in Fig.2. $V_{ip}(i)$ was computed as the variance of the distribution of $Sign(x_i)$ over 100 runs for an identical genotype, while $V_g(i)$ was computed as a variance of the distribution of $(\overline{Sign(x_i)})$ over 100 individuals, where $(\overline{Sign(x_i)})$ was the mean over 100 runs. The number of total genes was $N = 64$, while that of target genes was $k = 8$ (red $+$), 16(green $\times$), and 24 (blue $*$). The noise level was fixed at $\sigma = 0.1$. The initial density of non-zero elements in J_{ij} was 0.6. The plot of $(V_g(i)$ and $V_{ip}(i))$ for all genes i at the 400th generation. Here, the evolution to reach the genotype with the highest fitness value required more generations as the number of target genes was increased. (b) Relationship between $V_g(i)$ and $V_{ip}(i)$, for evolved GRNs that had a smaller fraction of non-zero elements in J_{ij}. The number of total genes was increased to $N = 200$, also to demonstrate that our results are independent of the number of genes. The variances were computed by the same procedure described in the above figures. The number of target genes was fixed at $k = 16$, and the noise level at $\sigma = 0.1$. The initial density of non-zero elements in J_{ij} was 0.05 (red $+$), 0.1 (green $\times$), and 0.2 (blue $*$), while the plots for higher non-zero fraction of J_{ij}'s also fitted well with the proportionality in this figure. The plot of $(V_g(i)$ and $V_{ip}(i))$ for all genes i at the 300th generation, when the fitness reached the highest value for most individuals.

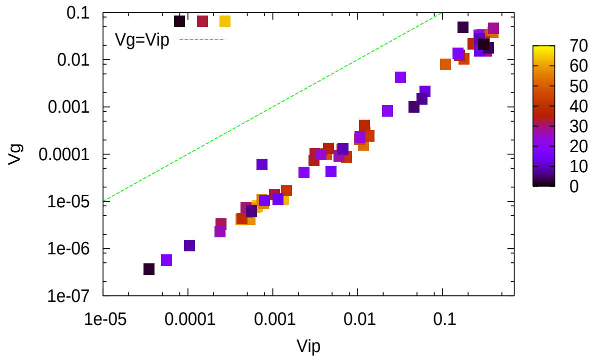

Fig. S5: Relationship between $V_g(i)$ and $V_{ip}(i)$ for the gene expression dynamics whose noise level depended on each gene. The noise level of each gene i, σ_i, was given by $0.05 + 0.1 \times i/N$ $(i = 1, 2, ..., N)$. To compute the variances, we used the same procedure adopted thus far. The number of total genes was $N = 64$, and the number of target genes was $k = 8$. The color represented the index of gene i, and accordingly, the noise level was σ_i. The initial density of non-zero elements in J_{ij} was 0.6. The plot of $(V_g(i)$ and $V_{ip}(i))$ for all genes i at the 200th generation. Note the proportionality between the 2 variances held true, while the noise level σ_i was not correlated with the phenotypic variances $V_{ip}(i)$ (or $V_g(i)$), because the color (showing the noise level for i) pattern was scrambled in the order of the magnitude of V_{ip} (or V_g). We studied several other forms of distribution of noise levels and confirmed that the proportionality is valid as long as the noise levels for most genes are higher than the threshold σ_c.

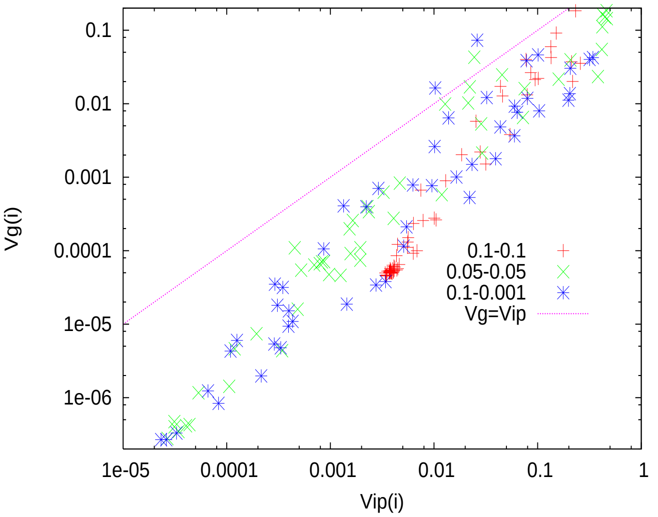

Fig. S6: Relationship between $V_g(i)$ and $V_{ip}(i)$ for a model with "extrinsic noise." In addition to the (intrinsic) noise applied to expression of each gene thus far, another source of noise $\zeta(t)$ that was common to all genes was applied. so that $dx_i/dt = \gamma(tanh[\beta \sum_j J_{ij}x_j] - x_j) + \sigma\eta_i(t) + \sigma^E\zeta(t)$, where $\eta_i(t)$ and $\zeta(t)$ were Gaussian white noise with $< \eta_i(t)\eta_j(t') >= \delta(t-t')\delta_{ij}$, and $< \zeta(t)\zeta(t') >= \delta(t-t')$. The noise $\eta_i(t)$ studied thus far took a different, uncorrelated value for each gene i. In this case again, there was a transition to robust evolution as σ was increased beyond σ_c. When $\sigma \ll \sigma_c$, the variances remained rather large even if the magnitude of extrinsic noise (σ^E) was large, and the 2 variances took similar values ($V_{ip} \sim V_g$) thereafter. Hence, it can be inferred that intrinsic noise plays a major role in decreasing the variance and increasing the robustness of phenotypes. When robustness was evolved, the proportionality between the 2 variances $V_{ip}(i)$ and $V_g(i)$ was again observed, as shown in this figure. The variances $V_g(i)$ and $V_{ip}(i)$ were computed in the manner described in the above figures. The overall proportionality between the 2 variances persisted beyond the robustness transition, while the deviation from the proportionality line was increased under the presence of the extrinsic (common) noise. The plot for $\sigma = \sigma^E = .1$ (red $+$), $\sigma = \sigma^E = 0.05$(green $\times$), and $\sigma = 0.001 < \sigma_c$, $\sigma^E = 0.1$(blue $*$). The variances were computed by the same procedure described before. The number of total genes was $N = 64$, and the number of target genes was $k = 8$. The initial density of non-zero elements in J_{ij} was 0.6. The plot of ($V_g(i)$ and $V_{ip}(i)$) for all genes i at the 200th generation.
